# PET motion correction using MR-derived motion parameters

**DOI:** 10.1186/2197-7364-1-S1-A53

**Published:** 2014-07-29

**Authors:** Matthew Bickell, Thomas Koesters, Fernando Boada, Johan Nuyts

**Affiliations:** Department of Nuclear Medicine, Medical Imaging Research Center, KU Leuven, Belgium; Center for Advanced Imaging Innovation and Research, New York University, Kragujevac, USA; Department of Radiology, NYU School of Medicine, New York, Bernard & Irene Schwartz Center for Biomedical Imaging, New York, USA

With the improving resolution of modern PET scanners, any slight motion during the scan can cause significant blurring and loss of resolution. MRI scanners have the capacity to perform quick successive scans and thus provide a means to track motion during a scan. Hence, with the advent of simultaneous PET-MR scanners, it has become possible to use the MR scanner to track the motion and thereby provide the necessary motion parameters to correct the PET data. Using a suitable segmentation approach a separate MR scan can provide the attenuation map to produce quantitative PET images.

An FDG brain scan was acquired on a Siemens Biograph mMR PET-MR scanner. The MR scan was acquired using the Golden Angle Radial Sparse Parallel (GRASP) sequence [1], simultaneously with a 5 minute PET scan, while the patient was asked to move his head repetitively from side to side for proof-of-principle purposes. A separate static scan was also acquired prior to the motion scan, to be used as a control. The MR data were divided into a series of 268 images with a frequency of approximately 1 Hz. The motion parameters were derived by performing a rigid (6 degrees-of-freedom) registration of the masked MR images to a chosen reference image. The PET list-mode data were corrected on an event-by-event basis [2, 3]. List-mode maximum likelihood expectation-maximisation (accelerated with ordered subsets [4]) was used for the reconstruction, incorporating the attenuation correction (as a pre-correction to the data) as well as weighted-average sensitivity [2] to achieve a quantitative reconstruction.

Motion correction successfully removed almost all motion artefacts, recovered the resolution and allowed for quantitative images to be produced. Techniques to improve upon the coarse sampling of the MR images, such as interpolating between motion data points, will be investigated.
